# Effects of luseogliflozin on suspected MASLD in patients with diabetes: a pooled meta-analysis of phase III clinical trials

**DOI:** 10.1007/s00535-024-02122-x

**Published:** 2024-07-26

**Authors:** Takumi Kawaguchi, Kenta Murotani, Hiromitsu Kajiyama, Hitoshi Obara, Hironori Yamaguchi, Yuko Toyofuku, Fumi Kaneko, Yutaka Seino, Saeko Uchida

**Affiliations:** 1 Division of Gastroenterology, Department of Medicine, Kurume University School of Medicine, 67 Asahi-Machi, Kurume, 830-0011 Japan; 2 Clinical Research Center, Kurume University Hospital, Kurume, Japan; 3 Biostatistics Center, Kurume University, Kurume, Japan; 4 School of Medical Technology, Kurume University, Kurume, Japan; 5 Taisho Pharmaceutical Co., Ltd, Tokyo, Japan; 6 Kansai Electric Power Hospital, Osaka, Japan

**Keywords:** Meta-analysis, Luseogliflozin, Sodium–glucose cotransporter 2 inhibitor, Metabolic dysfunction-associated steatotic liver disease, Diabetes mellitus

## Abstract

**Background:**

Luseogliflozin, a sodium–glucose cotransporter 2 inhibitor, potentially exerts pleiotropic effects on the liver. However, the sufficient evidence is still lacking. We aimed to investigate the effects of luseogliflozin on hepatic steatosis, fibrosis, and cardiometabolic risk factors in diabetic patients by a pooled meta-analysis.

**Methods:**

In this pooled meta-analysis, we enrolled diabetic patients who participated in phase III clinical trials of luseogliflozin (luseogliflozin group *n* = 302, placebo group *n* = 191). The primary outcomes were changes in fatty liver index (FLI) and Hepamet fibrosis score (HFS) after 24 weeks. The secondary outcomes were changes in cardiometabolic risk factors after 24 weeks. Statistical analysis was performed using propensity scoring analysis by the inverse probability of treatment weighting method.

**Results:**

Primary outcomes: Luseogliflozin significantly decreased FLI compared to placebo after 24 weeks (adjusted coefficient − 5.423, 95%CI − 8.760 to − 2.086, *P* = 0.0016). There was no significant difference in changes in HFS between the two groups. However, luseogliflozin significantly decreased HFS compared to placebo in diabetic patients with ALT > 30 U/L (adjusted coefficient − 0.039, 95%CI − 0.077 to − 0.001, *P* = 0.0438) and with FIB-4 index > 1.3 (adjusted coefficient − 0.0453, 95%CI − 0.075 to − 0.016, *P* = 0.0026). Secondary outcom8es: Luseogliflozin significantly decreased HbA1c level, HOMA-IR value, BMI, and uric acids level, and increased HDL cholesterol level compared to placebo.

**Conclusions:**

This pooled meta-analysis demonstrated that 24-week treatment with luseogliflozin improved hepatic steatosis and fibrosis indexes in diabetic patients, especially those with liver injury. Furthermore, luseogliflozin improved various cardiometabolic risk factors. Thus, luseogliflozin may be useful for improving MASLD in diabetic patients.

**Graphical abstract:**

**Supplementary Information:**

The online version supplementary material available at 10.1007/s00535-024-02122-x.

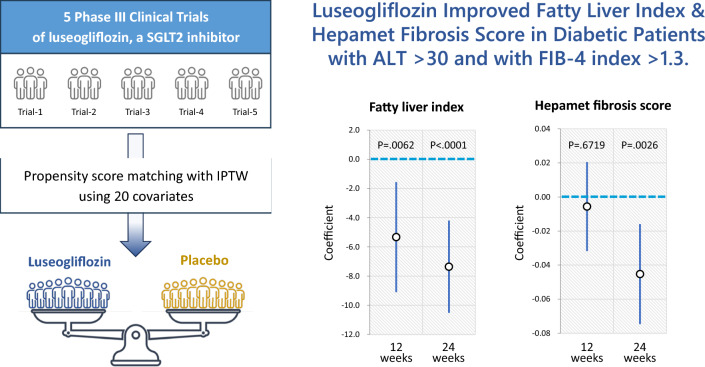

## Introduction

Hepatic steatosis is highly prevalent in patients with diabetes mellitus. American Diabetes Association recently updated the clinical practice recommendations and advocated the screening of non-alcoholic fatty liver disease (NAFLD) in patients with diabetes mellitus [1]. In the field of hepatology, the co-occurrence of hepatic steatosis and metabolic dysfunctions has also received a great deal of attention [2–4]. The NAFLD Nomenclature consensus group proposed to change the name of NAFLD to metabolic dysfunction-associated steatotic liver disease (MASLD) [2]. Hepatic steatosis and fibrosis increase the risk of liver cancer, major adverse cardiovascular events, and mortality in patients with diabetes mellitus [1, 5–7]. Therefore, it is important to consider a therapeutic strategy for improving hepatic steatosis and fibrosis in the management of patients with diabetes mellitus.

Sodium–glucose cotransporter 2 inhibitor (SGLT2i) is an anti-diabetic medication. Besides the glucose-lowering effect, SGLT2i exerts various pleiotropic effects including inhibition for the progression of heart failure and chronic kidney disease [8]. In addition, SGLT2i has been reported to exert beneficial effects on MASLD. Meta-analyses demonstrated that SGLT2i decreased serum levels of alanine aminotransferase (ALT) in patients with diabetes mellitus and MASLD [9, 10]. Furthermore, several studies reported the beneficial effects of SGLT2i on hepatic steatosis and fibrosis [11–15]. However, previous studies were conducted in a small number of subjects. In addition, these studies are single-arm trials [11–13] or open-label prospective studies with no background adjustment between the SGLT2i and control groups [14, 15]. Besides, previous meta-analyses reported conflicting results on the improvement of hepatic steatosis and fibrosis, which could be due to the differences in the study protocol of each clinical study [18, 19]. Additionally, the beneficial effects of SGLT2i on MASLD have been reported in studies with Western subjects [20–22] and there is limited evidence on the efficacy of SGLT2i in Japanese patients with MASLD. In fact, the evidence level for the recommendation of SGLT2i to diabetic patients with NAFLD/NASH is still limited (Evidence Level C, Strength 2) in the evidence-based clinical practice guidelines for NAFLD/NASH 2020 [23, 24]. Thus, the effects of SGLT2i on hepatic steatosis and fibrosis have not reached a consensus and further research is needed.

Luseogliflozin is a unique SGLT2i. Different from other SGLT2i, luseogliflozin is metabolized by cytochrome P450-mediated oxidation in the liver and kidney and by glucuronide conjugate in the liver and small intestine [25]. In other words, luseogliflozin has multiple drug metabolism pathways in the kidney and small intestine. A clinical pharmacokinetic study for patients with mild/moderate hepatic impairment demonstrated that luseogliflozin can be safely administered to cirrhotic patients with Child–Pugh class B [26]. These studies suggest that luseogliflozin is safe and effective in patients with chronic liver disease. In addition, a few single-arm trials demonstrated that luseogliflozin significantly improved hepatic steatosis and hepatic fibrosis in patients with diabetes mellitus and MASLD [11–13]. However, these previous studies were exploratory trials with small sample sizes and no placebo group. To date, 5 phase III clinical trials conducted to investigate the safety and efficacy of luseogliflozin in patients with diabetes mellitus. These data have not been used for verifying the effects of luseogliflozin on hepatic steatosis and fibrosis.

The aim of this study is to investigate the effects of luseogliflozin on hepatic steatosis and fibrosis by a pooled meta-analysis of phase III clinical trials. We also investigated the effects of luseogliflozin on cardiometabolic risk factors and hepatic inflammation in diabetic patients.

## Subjects and methods

### Study design and ethics

This is a pooled meta-analysis aiming to investigate the effects of luseogliflozin on hepatic steatosis and fibrosis indexes, cardiometabolic risk factors, and hepatic inflammation in Japanese patients with diabetes mellitus. All phase III clinical trials were registered with Clinical Trials.gov (http://www.clinicaltrials.gov) and conducted according to the Declaration of Helsinki and Good Clinical Practice Guidelines. All participants provided prior written consent before the onset of the clinical trials. The protocol of this pooled meta-analysis was approved by the Institutional Review Board of Kurume University School of Medicine (No. 23024). Personal information was protected during data collection.

### Data source

Data were pooled from 5 phase III clinical trials that examined the safety and efficacy of luseogliflozin in patients with diabetes mellitus in Japan [27–30] (Supplementary Table S1).

### Inclusion and exclusion criteria

We enrolled patients with diabetes who participated in phase III clinical trials of luseogliflozin. The inclusion criteria of the clinical trials were (1) > 20 years old and (2) HbA1c ranged from 6.9% to 10.5%. Exclusion criteria of the clinical trials were (1) AST or ALT level > 2.5 times the upper limit of reference values and (2) patients who did not meet the criteria for concomitant use of diabetes medications in each clinical trial (Supplementary Table 1).

In this pooled meta-analysis, we further excluded patients with any of the following coexisting diseases that may affect hepatic steatosis and fibrosis: any type of malignancy, hepatitis virus-related liver disease, alcohol-related liver disease, autoimmune liver disease, hemochromatosis, drug-induced liver injury, alcoholic-related pancreas disease, alcoholism, chronic pancreatitis, intraductal papillary mucinous neoplasia, Basedow’s disease, major depressive disorder, and schizophrenia.

### Patient selection

**Fig. 1:**
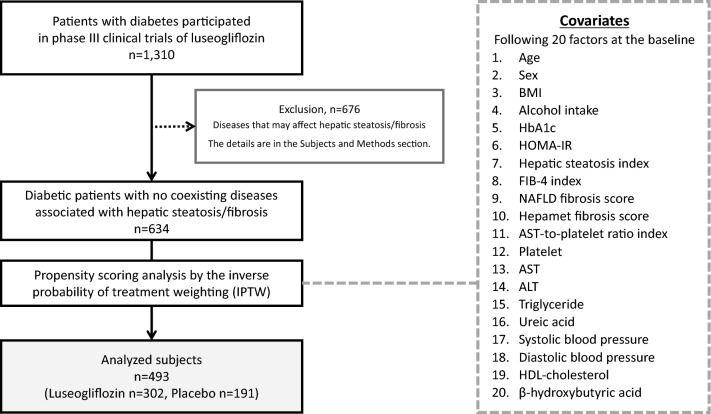
Flow diagram of the patient selection process

The patient selection process is summarized in Fig. 1. We enrolled a total of 1,310 patients with diabetes in 5 phase III clinical trials of luseogliflozin. Then, we excluded 676 patients due to coexisting diseases that may affect hepatic steatosis and fibrosis. The remaining 634 patients were subjected to propensity scoring analysis by the inverse probability of treatment weighting using the 20 covariate factors at the baseline. Finally, we analyzed a total of 493 patients with diabetes with matched covariates between the two groups (Luseogliflozin group, *n* = 302; Placebo group, *n* = 191).

### Assessment for hepatic steatosis and fibrosis

Hepatic steatosis was assessed by three non-invasive tests, such as fatty liver index, NAFLD liver fat score, and hepatic steatosis index [31] before, 12 weeks, and 24 weeks after the treatment. Hepatic fibrosis was assessed by four non-invasive tests, such as Hepamet fibrosis score, aspartate aminotransferase (AST)-to-platelet ratio index (APRI), FIB-4 index, and NAFLD fibrosis score[32] before, 12 weeks, and 24 weeks after the treatment.

### Cardiometabolic risk factors

We assessed the following cardiometabolic risk factors before, 12 weeks, and 24 weeks after the treatment: hemoglobin A1c (HbA1c), homeostasis model assessment of insulin resistance (HOMA-IR), body mass index (BMI), high-density lipoprotein (HDL) cholesterol, low-density lipoprotein (LDL) cholesterol, triglyceride, and uric acids.

### Primary and secondary outcomes

The primary outcomes were changes in hepatic steatosis indexes and fibrosis indexes 12 weeks and 24 weeks after treatment. The secondary outcomes were changes in HbA1c, HOMA-IR, BMI, HDL cholesterol, LDL cholesterol, triglyceride, and uric acids 12 weeks and 24 weeks after treatment. In addition, we evaluated changes in the following liver function tests as the secondary outcomes: AST, ALT, γ-glutamyl transpeptidase (γ-GT), albumin, and platelet count.

### Statistical analysis

Continuous variables are expressed as median, interquartile range, and range. Categorical variables are expressed as percentages and numbers. All statistical analyses were performed by biostatisticians (K.M. and H.O.).

To reduce the impact of treatment selection bias and potential confounders in this study, we employed propensity scoring analysis by the inverse probability of treatment weighting method to adjust for clinically associated factors. A logistic regression model involving 20 covariates at the baseline was used to estimate the propensity score; age, sex, BMI, alcohol intake, HbA1c, HOMA-IR, hepatic steatosis index, FIB-4 index, NAFLD fibrosis score, Hepamet fibrosis score, APRI, platelet, AST, ALT, triglyceride, uric acid, systolic blood pressure, diastolic blood pressure, HDL cholesterol, and β-hydroxybutyric acid.

The differences between the Luseogliflozin and Placebo groups were calculating regression coefficient and 95% confidence intervals (CI) using a propensity score-weighted linear regression model. We also performed stratification analysis according to ALT > 30 U/L and FIB-4 index > 1.3. Two-sided *P* < 0.05 was considered to indicate statistical significance. All statistical analyses were conducted using SAS 9.4 software (SAS Institute Inc, Cary, NC, USA).

## Results

### Patients’ characteristics

The baseline characteristics of all patients are summarized in Table 1. In the Luseogliflozin group, the number of patients was 302 and all patients were Asian with a median age of 63 years. The male rate was 74.8% and the median BMI was 24.6. Median HbA1c and HOMA-IR were 7.8% and 2.2, respectively.

**Table 1:** Baseline characteristics of all patients

	Placebo			Luseogliflozin		
	Median (IQR)	Range		Median (IQR)	Range	P
N	191	N/A		302	N/A	
Race (Asian/non-Asian)	100%/0% (191/0)			100%/0% (302/0)		
Age (years)	62 (56–69)	30–86		63 (56–70)	31–89	0.6323
Sex (Male/female)	71.2%/28.8% (136/55)	N/A		74.8%/25.2% (226/76)	N/A	0.3740
BMI	24.6 (22.4–27.2)	17.4–40.5		24.6 (22.9–27.3)	16.0–53.4	0.6343
HbA1c (%)	7.9 (7.4–8.4)	6.7–10.7		7.8 (7.4–8.4)	6.8–11.0	0.5757
HOMA-IR	2.3 (1.6–3.6)	0.3–12.1		2.2 (1.4–3.3)	0.3–20.4	0.3829
Systolic blood pressure (mmHg)	130 (121–138)	93–169		130 (120–140)	94–178	0.6363
Diastolic blood pressure (mmHg)	76 (70–82)	52–97		75 (70–81)	47–104	0.2491
Fatty liver index	36.4 (22.0–61.0)	3.9–98.5		45.6 (24.3–69.7)	9.7–99.5	0.1438
NAFLD liver fat score	− 0.1 (− 1.3–0.8)	− 2.7–3.4		0.1 (− 0.9–1.0)	− 2.8–5.5	0.1889
Hepatic steatosis index	35.6 (32.0–38.9)	25.0–56.0		35.3 (32.0–39.2)	23.1–66.4	0.7421
Hepamet fibrosis score	0.2 (0.1–0.3)	0.0–0.7		0.2 (0.1–0.2)	0.0–0.8	0.9704
APRI	0.30 (0.24–0.39)	0.10–0.93		0.30 (0.23–0.39)	0.06–1.17	0.4263
FIB-4 index	1.5 (1.2–1.9)	0.5–5.6		1.6 (1.2–2.1)	0.3–5.9	0.3906
NAFLD fibrosis score	− 0.463 (− 1.046–0.168)	− 3.371–2.002		− 0.369 (− 1.025–0.285)	− 8.801–3.336	0.3164
Platelet count (× 10^4^/μL)	20.4 (17.3–23.5)	9.2–39.8		19.8 (17.5–22.7)	7.1–86.1	0.3539
AST (IU/L)	25 (20–30)	14–84		23 (20–30)	14–68	0.3170
ALT (IU/L)	24 (18–33)	10–99		24 (16–33)	7–96	0.2153
GGT (IU/L)	31 (22–53)	9–218		34 (23–54)	6–221	0.4366
Albumin (g/dL)	4.4 (4.2–4.6)	3.7–5.3		4.4 (4.3–4.6)	3.4–5.2	0.8098
Creatinine (mg/dL)	0.8 (0.6–0.9)	0.4–1.7		0.8 (0.6–1.0)	0.4–2.0	0.0145
eGFR (mL/min/1.73m^2^)	78.3 (62.3–93.0)	33.1–143.4		71.2 (58.0–88.1)	29.4–142.0	0.0193
HDL-cholesterol (mg/dL)	54 (44–66)	27–120		54 (46–65)	24–108	0.4599
LDL-cholesterol (mg/dL)	118 (100–137)	45–239		120 (101–140)	40–222	0.8926
Triglyceride (mg/dL)	133 (86–181)	30–810		126 (88–178)	31–656	0.6125
Ureic acid (mg/dL)	5.3 (4.3–6.3)	2.5–8.5		5.4 (4.4–6.2)	0.7–10.3	0.9570
β-hydroxybutyrate (μmol/L)	51.0 (28.8–78.7)	13.2–699.0		55.3 (34.5–99.9)	10.3–507.0	0.0309
Medication						
Biguanide	12.6% (24/191)	N/A		10.9% (33/302)	N/A	0.5795
α-glucosidase inhibitor	5.2% (10/191)	N/A		5.6% (17/302)	N/A	0.8516
Thiazolidinedione	6.3% (12/191)	N/A		8.3% (25/302)	N/A	0.4890
DPP4 inhibitor	9.4% (18/191)	N/A		11.6% (35/302)	N/A	0.4496
Glinide	2.6% (5/191)	N/A		2.6% (8/302)	N/A	0.9832
Sulfonylurea	49.7% (95/191)	N/A		64.6% (195/302)	N/A	0.0013
GLP-1 receptor agonist	0.0% (0/191)	N/A		0.0% (0/302)	N/A	N/A
Glimin	0.0% (0/191)	N/A		0.0% (0/302)	N/A	N/A
Exogenous insulin	0.5% (1/191)	N/A		0.7% (2/302)	N/A	0.8470
ACE	3.1% (6/191)	N/A		6.3% (19/302)	N/A	0.1204
ARB	42.4% (81/191)	N/A		42.1% (127/302)	N/A	0.9380
Direct renin inhibitor	0.0% (0/191)	N/A		1.7% (5/302)	N/A	0.0739
Lipid-lowering medication	44.0% (84/191)	N/A		49.3% (149/302)	N/A	0.2456

*IQR* interquartile range; *N/A* not applicable; *BMI* body mass index; *HbA1c* hemoglobin A1c; *HOMA-IR* homeostasis model assessment of insulin resistance; *NAFLD* non-alcoholic fatty liver disease; *APRI* aspartate aminotransferase-to-platelet ratio index; *AST* aspartate aminotransferase; *ALT* alanine aminotransferase; *GGT* γ-glutamyl transpeptidase; *eGFR* estimated glomerular filtration rate; *HDL* high-density lipoprotein; *LDL* low-density lipoprotein; *DPP4* dipeptidyl-peptidase 4; *GLP-1* glucagon-like peptide-1; *ACE* angiotensin-converting enzyme; *ARB* angiotensin receptor blocker

The median fatty liver index, NAFLD liver fat score, and hepatic steatosis index were 45.6, 0.1, and 35.3, respectively. The median Hepamet fibrosis score, APRI, FIB-4 index, and NAFLD fibrosis score were 0.2, 0.3, 1.6, and − 0.369, respectively. The median serum levels of HDL-cholesterol, LDL-cholesterol, triglyceride, and uric acid were 54 mg/dL, 120 mg/dL, 126 mg/dL, and 5.4 mg/dL, respectively. These data for the Luseogliflozin group were not significantly different from those for the Placebo group. In addition, there was no significant difference in liver function tests and prevalence of those on most anti-diabetic medications except for sulfonylurea. No significant difference was observed in the use of antihypertensive and lipid-lowering medication between the two groups.

The baseline characteristics of patients with ALT > 30 U/L and FIB-4 index > 1.3 were summarized in Supplementary Tables S2 and S3, respectively. Similar to the results in all subjects, there were no significant differences between the Luseogliflozin and Placebo groups for most of the variables in both subjects with ALT > 30 U/L and subjects with FIB-4 index > 1.3.

### Primary outcomes

#### Analysis using all subjects

**Fig. 2:**
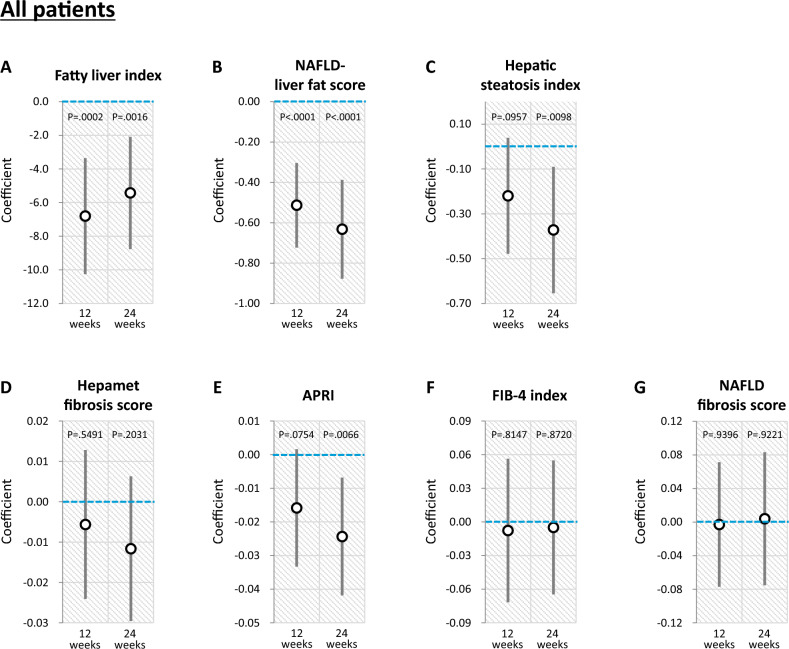
Effects of luseogliflozin on hepatic steatosis and fibrosis indexes after 12 and 24 weeks in all patients. **A** Fatty liver index, **B** NAFLD liver fat score, **C** Hepatic steatosis index, **D** Hepamet fibrosis score, **E** APRI, **F** FIB-4 index, **G** NAFLD fibrosis score. *NAFLD* non-alcoholic fatty liver disease; *APRI* aspartate aminotransferase-to-platelet ratio index

Luseogliflozin significantly decreased fatty liver index and NAFLD liver fat score compared to placebo after 12 and 24 weeks (Fig. 2A and 2B). Luseogliflozin also significantly decreased hepatic steatosis index and APRI compared to placebo after 24 weeks (Fig. 2C and 2E).

No significant change was observed in Hepamet fibrosis score, APRI, FIB-4 index, and NAFLD fibrosis score between the Luseogliflozin and Placebo groups after 12 and 24 weeks (Figs. 2D, 2F, and 2G). Luseogliflozin significantly decreased APRI after 24 weeks (Figs. 2E).

#### Stratification analysis according to ALT > 30 U/L

**Fig. 3:**
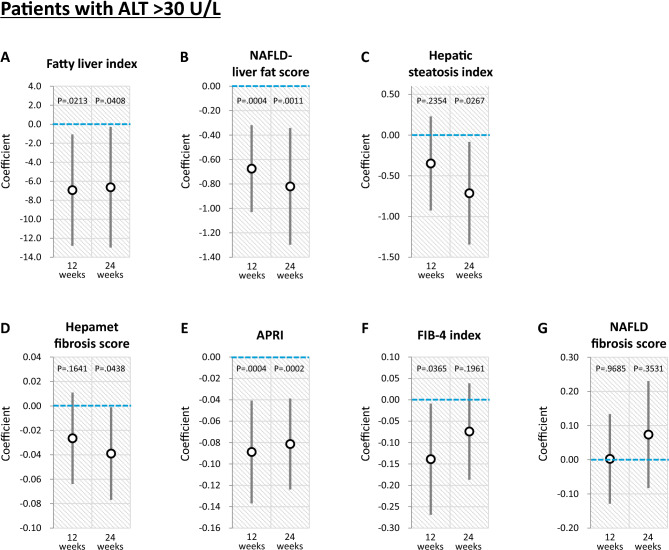
Effects of luseogliflozin on hepatic steatosis and fibrosis indexes after 12 and 24 weeks in patients with ALT > 30 U/L. **A** Fatty liver index, **B** NAFLD liver fat score, **C** Hepatic steatosis index, **D** Hepamet fibrosis score, **E** APRI, **F** FIB-4 index, **G** NAFLD fibrosis score. *NAFLD* non-alcoholic fatty liver disease; *APRI* aspartate aminotransferase-to-platelet ratio index

In a stratification analysis according to ALT > 30 U/L, luseogliflozin significantly decreased fatty liver index, NAFLD liver fat score, and hepatic steatosis index after 24 weeks (Figs. 3A, 3B, and 3C). In addition, luseogliflozin significantly decreased Hepamet fibrosis score and APRI after 24 weeks (Fig. 3D and 3E). In contrast, no significant change was observed in FIB-4 index and NAFLD fibrosis score between the Luseogliflozin and Placebo groups after 24 weeks (Fig. 3F and 3G).

#### Stratification analysis according to FIB-4 index > 1.3

**Fig. 4:**
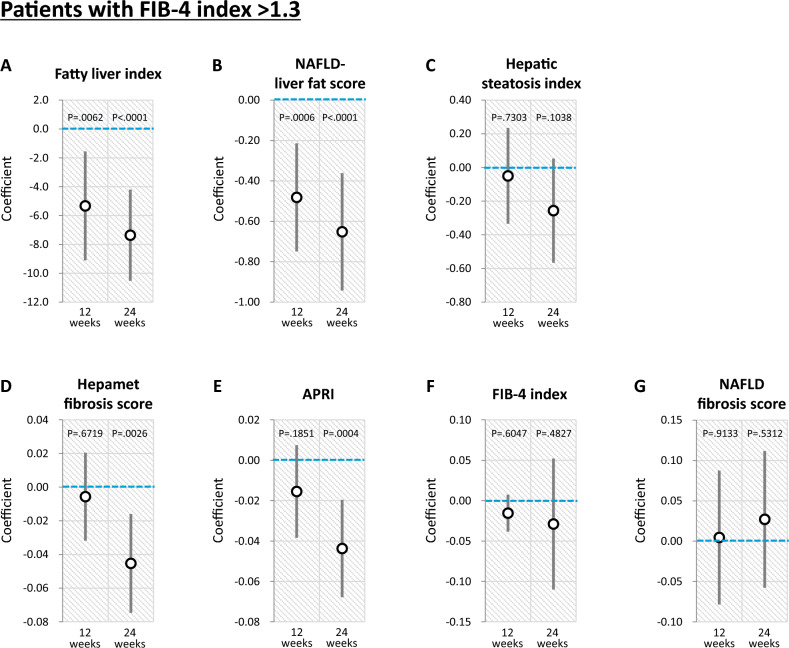
Effects of luseogliflozin on hepatic steatosis and fibrosis indexes after 12 and 24 weeks in patients with FIB-4 index > 1.3. **A** Fatty liver index, **B** NAFLD liver fat score, **C** Hepatic steatosis index, **D** Hepamet fibrosis score, **E** APRI, **F** FIB-4 index, **G** NAFLD fibrosis score. *NAFLD* non-alcoholic fatty liver disease; *APRI* aspartate aminotransferase-to-platelet ratio index

In a stratification analysis according to FIB-4 index > 1.3, luseogliflozin significantly decreased fatty liver index and NAFLD liver fat score after 12 and 24 weeks (Fig. 4A and 4B). In contrast, no significant change was observed in hepatic steatosis index between the Luseogliflozin and Placebo groups after 12 and 24 weeks (Fig. 4C).

Luseogliflozin significantly decreased Hepamet fibrosis score and APRI after 24 weeks (Fig. 4D and 4E). In contrast, no significant change was observed in FIB-4 index and NAFLD fibrosis score between the Luseogliflozin and Placebo groups after 24 weeks (Figs. 4F and 4G).

### Secondary outcomes

#### Analysis using all subjects

Luseogliflozin significantly decreased HbA1c level and HOMA-IR level compared to placebo after 12 and 24 weeks (Table 2). In addition, luseogliflozin significantly decreased BMI and uric acid levels, and increased HDL cholesterol levels compared to placebo after 12 and 24 weeks.

**Table 2:** Changes in cardiometabolic risk factors in all patients, patients with ALT > 30 U/L, and with FIB-4 index > 1.3

	All patients				ALT > 30 U/L				FIB-4 index > 1.3			
	Adjusted Coefficient	95% CI	95% CI	P	Adjusted Coefficient	95% CI	95% CI	P	Adjusted Coefficient	95% CI	95% CI	P
		high	low			high	low			high	low	
HbA1c												
12 weeks	− 0.581	− 0.674	− 0.489	< .0001	− 0.82	− 1.029	− 0.611	< .0001	− 0.662	− 0.785	− 0.539	< .0001
24 weeks	− 0.629	− 0.755	− 0.503	< .0001	− 0.796	− 1.079	− 0.512	< .0001	− 0.781	− 0.958	− 0.603	< .0001
HOMA-IR												
12 weeks	− 0.745	− 0.995	− 0.496	< .0001	− 1.278	− 1.883	− 0.673	< .0001	− 0.815	− 1.137	− 0.492	< .0001
24 weeks	− 0.77	− 1.031	− 0.5 − 1	< .0001	− 1.377	− 2.035	− 0.719	< .0001	− 0.903	− 1.235	− 0.572	< .0001
BMI												
12 weeks	− 0.517	− 0.429	− 0.604	< .0001	− 0.42	− 0.744	− 0.582	< .0001	− 0.294	− 0.51	− 0.402	< .0001
24 weeks	− 0.536	− 0.416	− 0.657	< .0001	− 0.331	− 0.806	− 0.569	< .0001	− 0.212	− 0.527	− 0.369	< .0001
HDL-cholesterol												
12 weeks	3.04	0.133	1.586	0.0325	3.279	− 2.178	0.551	0.6905	3.989	0.346	2.168	0.0198
24 weeks	5.529	2.852	4.191	< .0001	5.016	− 0.045	2.485	0.0542	7.102	3.793	5.448	< .0001
LDL-cholesterol												
12 weeks	2.397	− 4.876	− 1.239	0.5034	6.689	− 7.795	− 0.553	0.8802	2.472	− 6.198	− 1.863	0.3985
24 weeks	5.106	− 2.332	1.387	0.464	11.281	− 5.55	2.865	0.5018	4.707	− 3.872	0.417	0.8484
Triglyceride												
12 weeks	5.915	− 21.31	− 7.697	0.2671	27.38	− 42.855	− 7.737	0.6638	20.065	− 11.323	4.371	0.5841
24 weeks	0.83	− 25.085	− 12.128	0.0665	1.028	− 48.435	− 23.703	0.0602	4.471	− 25.837	− 10.683	0.1664
Uric acids												
12 weeks	− 0.119	− 0.369	− 0.244	0.0001	0.05	− 0.446	− 0.198	0.1165	− 0.15	− 0.439	− 0.294	< .0001
24 weeks	− 0.202	− 0.478	− 0.34	< .0001	0.118	− 0.434	− 0.158	0.2603	− 0.143	− 0.461	− 0.302	0.0002

*ALT* alanine aminotransferase; *CI* confidence interval; *HbA1c* hemoglobin A1c; *HOMA-IR* homeostasis model assessment of insulin resistance; *BMI* body mass index; *HDL* high-density lipoprotein; *LDL* low-density lipoprotein

Luseogliflozin significantly decreased AST, ALT, and γ-GT levels and increased albumin levels after 24 weeks (Table 3). In contrast, no significant difference was seen in platelet count between the Luseogliflozin and Placebo groups after 12 and 24 weeks.

**Table 3:** Changes in liver function tests in all patients, patients with ALT > 30 U/L, and with FIB-4 index > 1.3

	All patients				ALT > 30 U/L				FIB-4 index > 1.3	
	Adjusted Coefficient	95% CI	95% CI	P	Adjusted Coefficient	95% CI	95% CI	P	Adjusted Coefficient	95% CI
		high	low			high	low			high
AST										
12 weeks	0.114	− 2.273	− 1.079	0.0761	− 2.476	− 8.899	− 5.688	0.0006	0.658	− 2.285
24 weeks	− 0.739	− 3.369	− 2.054	0.0023	− 2.821	− 9.556	− 6.188	0.0004	− 1.301	− 4.293
ALT										
12 weeks	− 0.107	− 3.66	− 1.884	0.0378	− 3.633	− 11.735	− 7.684	0.0003	1.669	− 2.934
24 weeks	− 1.524	− 5.323	− 3.424	0.0004	− 5.051	− 14.588	− 9.82	< .0001	− 1.407	− 5.502
GGT										
12 weeks	− 2.957	− 10.799	− 6.878	0.0006	− 8.273	− 21.848	− 15.06	< .0001	0.918	− 9.605
24 weeks	− 3.842	− 11.478	− 7.66	< 0.0001	− 1.434	− 18.867	− 10.15	0.0228	− 2.063	− 11.549
Albumin										
12 weeks	0.047	− 0.039	0.004	0.8472	0.07	− 0.094	− 0.012	0.7712	0.034	− 0.073
24 weeks	0.087	0.001	0.044	0.0472	0.104	− 0.066	0.019	0.6597	0.08	− 0.024
Platelet										
12 weeks	0.649	− 0.231	0.209	0.3511	1.208	− 0.405	0.401	0.3272	0.671	− 0.227
24 weeks	0.518	− 0.426	0.046	0.8483	0.886	− 1.185	− 0.149	0.7757	0.425	− 0.504

*ALT* alanine aminotransferase; *CI* confidence interval; *AST* aspartate aminotransferase; *GGT*, γ-glutamyl transpeptidase

#### Stratification analysis according to ALT > 30 U/L

In a stratification analysis according to ALT > 30 U/L, luseogliflozin significantly decreased HbA1c level and HOMA-IR level compared to placebo after 12 and 24 weeks (Table 2). In addition, luseogliflozin significantly decreased BMI after 12 and 24 weeks.

Luseogliflozin significantly decreased AST, ALT, and γ-GT levels after 12 weeks and 24 weeks (Table 3). In contrast, no significant difference was seen in albumin level and platelet count between the Luseogliflozin and Placebo groups after 12 and 24 weeks.

#### Stratification analysis according to FIB-4 index > 1.3

In a stratification analysis according to FIB-4 index > 1.3, luseogliflozin significantly decreased HbA1c level and HOMA-IR level compared to placebo after 12 and 24 weeks (Table 3). In addition, luseogliflozin significantly decreased BMI and uric acid levels, and increased HDL cholesterol levels compared to placebo after 12 and 24 weeks.

Luseogliflozin significantly decreased AST, ALT, and γ-GT levels after 24 weeks (Table 3). In contrast, no significant difference was seen in albumin level and platelet count between the Luseogliflozin and Placebo groups after 12 and 24 weeks.

## Discussion

This pooled meta-analysis demonstrated that 24-week treatment with luseogliflozin improved hepatic steatosis and fibrosis indexes in diabetic Japanese patients, especially those with liver injury by comparing to a background-adjusted placebo group. Furthermore, luseogliflozin improved various cardiometabolic risk factors including not only HbA1c level but also HOMA-IR, BMI, HDL-cholesterol, and uric acids. Luseogliflozin also improved serum markers for hepatic inflammation.

SGLT2i has been reported to improve hepatic steatosis in single-arm trials [11–13], an open-label prospective study [14], and a meta-analysis of randomized controlled trials [33]. In addition, our pooled meta-analysis contributed to the further accumulation of the evidence. A possible mechanism for SGLT2i-induced improvement of steatosis is a reduction of insulin resistance and BMI [34] as observed in our study. Furthermore, we previously reported that expression of SGLT2 is observed in polarized human liver cancer cell lines and human liver tissue, and SGLT2i up-regulates AMP-activated protein kinase (AMPK) in hepatocytes [35, 36]. Chen et al. demonstrated that an activation of AMPK promotes fatty acid oxidation and mitochondrial biogenesis in hepatocytes leading to amelioration of hepatic steatosis [37]. Chun et al. also demonstrated increased SGLT2 expression of hepatocytes in patients with MASLD and SGLT2i alleviated hepatic steatosis through autophagy activation in hepatocytes [38]. Thus, SGLT2i may improve hepatic steatosis by both indirect and direct mechanisms to the liver.

SGLT2i has been reported to have the potential to improve hepatic fibrosis in a retrospective study [16] and randomized controlled trials [15, 17]. However, meta-analyses reported conflicting results on the improvement of hepatic fibrosis, which could be due to the differences in the study protocol of each clinical study [18, 19]. We first conducted a pooled meta-analysis using phase III clinical trials of luseogliflozin with identical protocols. Our study provided a higher level of evidence for the beneficial effects of SGLT2i on hepatic fibrosis indexes in Japanese patients with diabetes and ALT > 30 U/L or FIB-4 index > 1.3. Shimizu et al. and Arai et al. previously reported that SGLT2i significantly improved liver stiffness evaluated by transient elastography in diabetic patients with NAFLD [39, 40]. Furthermore, Akuta et al. demonstrated an improvement in hepatic steatosis and fibrosis evaluated by liver biopsy 24 weeks after treatment with SGLT2i in patients with NAFLD and type 2 diabetes mellitus [16, 41, 42]. Along with these previous reports, our study may increase the evidence level for SGLT2i in the guidelines and may contribute to improving MASLD in daily clinical practice.

Since metabolic dysfunctions are risk factors for hepatic fibrosis [4], a possible mechanism for SGLT2i-induced improvement of hepatic fibrosis is an amelioration of various cardiometabolic risk factors as observed in this study. In addition, Wakamatsu et al. demonstrated that luseogliflozin ameliorated hepatic fibrosis via decreasing hepatic inflammation through suppression of monocyte chemotactic protein-1 in a model mouse for MASH [43]. Since significant reductions in AST and ALT levels were observed in this study, amelioration of hepatic inflammation may contribute to SGLT2i-induced improvement of hepatic fibrosis indexes. Moreover, Shen et al. recently reported that SGLT2i inhibits the transforming growth factor-β pathway by downregulating miR-34a-5p in hepatic stellate cells, leading to amelioration of hepatic fibrosis [44]. Thus, SGLT2i may improve hepatic fibrosis by regulating metabolic dysfunction, hepatic inflammation, and hepatic stellate cells.

Since we could not evaluate liver biopsy, we employed four non-invasive indexes for hepatic fibrosis. It remains unclear which index is suitable for the evaluation of changes in hepatic fibrosis. However, previous studies examined the dynamic response of non-invasive indexes to histological changes using paired liver biopsies. The previous studies found that APRI, FIB-4 index, and NAFLD fibrosis score poorly respond to the improvement of hepatic fibrosis [45–47]. In contrast, Vilar-Gomez et al. reported that changes in HbA1c, platelets, and ALT normalization better predict the improvement of hepatic fibrosis than APRI, FIB-4 index, and NAFLD fibrosis score [45]. Moreover, Koo et al. reported that HOMA-IR values responded to changes in hepatic fibrosis status in patients with MASLD [48]. Hepamet fibrosis score includes these factors that responded to the improvement of hepatic fibrosis [32]. In fact, Hepamet fibrosis score has been reported to respond to a histological improvement of hepatic fibrosis in patients with MASLD who underwent bariatric surgery [49]. Taken together, Hepamet fibrosis score seems to be a suitable index for evaluating changes in hepatic fibrosis. Therefore, the results of this analysis suggest that hepatic fibrosis may be improved 24 weeks after treatment with luseogliflozin in diabetic patients with ALT > 30 U/L and with FIB-4 index > 1.3. In addition, a pharmacokinetic study for type 2 diabetes patients with mild/moderate hepatic impairment demonstrated that there was no obvious proportionality between the parameters of impaired hepatic function and a greater exposure to luseogliflozin, especially in area under the curve, which represents pharmacological effects [26]. This could be because luseogliflozin is metabolized or eliminated by multiple pathways [25]. These studies suggest that luseogliflozin is safe and effective in patients with chronic liver disease.

There are some limitations in this study. First, all of the phase III clinical trials were conducted in Japan. Second, the assessment of hepatic steatosis and fibrosis was based on non-invasive biological tests. Third, the treatment period was 24 weeks. Thus, further study should be designed as a meta-analysis of clinical trials that were conducted in different regions and include information for imaging modalities or liver biopsy and with longer treatment periods.

In conclusion, we performed a pooled meta-analysis using 5 phase III clinical trials conducted in Japan. We demonstrated that 24-week treatment with luseogliflozin improved hepatic steatosis and fibrosis indexes in diabetic Japanese patients, especially those with liver injury by comparing to a background-adjusted placebo group. Moreover, luseogliflozin improved various cardiometabolic risk factors and hepatic inflammatory markers. Thus, luseogliflozin may be beneficial for improving MASLD in patients with diabetes.

### Supplementary Information

Below is the link to the electronic supplementary material.

Supplementary material 1 (DOCX 32 KB)
