# Supplementary material for: Effects of luseogliflozin on suspected MASLD in patients with diabetes: a pooled meta-analysis of phase III clinical trials
Source: J Gastroenterol. 2024 Jul 26;59(9):836–48. doi: 10.1007/s00535-024-02122-x (PMC11338969; doi:10.1007/s00535-024-02122-x)
Supplement: Supplementary file 1 — Supplementary material 1 (DOCX 32 KB) [file 535_2024_2122_MOESM1_ESM.docx]

Supplementary Table 1. Characteristics of phase III clinical trials used in this pooled meta-analysis

|  | **Phase III**  **clinical traial-1** | **Phase III**  **clinical traial-2** | **Phase III**  **clinical traial-3** | **Phase III**  **clinical traial-4** | **Phase III**  **clinical traial-5** |
| --- | --- | --- | --- | --- | --- |
| Study design | Open-label,  single arm | Open-label,  single arm | Randomized controlled trial | Randomized controlled trial | Randomized controlled trial |
| Inclusion criteria | >20 years old, HbA1c 6.9%-10.5% | | | | |
| Exclusion criteria | AST or ALT level >2.5 times the upper limit of reference value | | | | |
| n | 299 | 487 | 221 | 145 | 158 |
| Dose of luseogliflozin | 2.5 mg/day | 2.5 mg/day | 2.5 mg/day | 2.5 mg/day | 2.5 mg/day |
| Administration period of luseogliflozin | 52 weeks | 52 weeks | 52 weeks | 52 weeks | 24 weeks |
| Concomitant medications for diabetes mellitus | Concomitant medications prohibited | Up to 1 other medication allowed | Sulfonylurea allowed | Up to 2 other medications allowed | Concomitant medications prohibited |
| Glucagon-like peptide-1 agonist | No | No | No | No | No |

Abbreviations: HbA1c, hemoglobin A1c; AST, aspartate aminotransferase; ALT, alanine aminotransferase.

Supplementary Table 2. Baseline characteristics of subjects with ALT >30 U/L

|  | Placebo | |  | Luseogliflozin | |  |
| --- | --- | --- | --- | --- | --- | --- |
|  | Median (IQR) | Range |  | Median (IQR) | Range | P |
| n | 57 | N/A |  | 86 | N/A |  |
| Age (years) | 58 (51—66) | 34—76 |  | 59 (52—67) | 36—80 | 0.6559 |
| Sex (Male/female) | 73.7%/26.3%  (42/15) | N/A |  | 77.9%/22.1%  (67/19) | N/A | 0.5614 |
| BMI | 26.0 (23.7—27.9) | 18.9—40.5 |  | 26.6 (23.9—30.0) | 19.6—53.4 | 0.2746 |
| HbA1c (%) | 7.9 (7.3—8.3) | 6.9—10.2 |  | 8.0 (7.5—8.8) | 6.8—10.8 | 0.1018 |
| HOMA-IR | 3.4 (2.3—5.0) | 0.6—11.1 |  | 3.3 (2.1—5.0) | 0.8—20.4 | 0.8528 |
| Systolic blood pressure (mmHg) | 128 (119—135) | 100—154 |  | 131 (121—140) | 98—178 | 0.2708 |
| Diastolic blood pressure (mmHg) | 78 (71—83) | 58—97 |  | 77 (70—85) | 55—104 | 0.8478 |
| Fatty liver index | 57.4 (33.8—69.3) | 9.6—98.5 |  | 67.2 (40.0—80.1) | 9.7—99.5 | 0.3222 |
| NAFLD liver fat score | 0.9 (0.0—2.4) | -1.1—3.3 |  | 1.0 (0.2—1.7) | -1.4—5.5 | 0.9317 |
| Hepatic steatosis index | 38.2 (36.3—47.5) | 27.2—56.0 |  | 39.3 (36.6—66.4) | 31.3—66.4 | 0.2551 |
| Hepamet fibrosis score | 0.2 (0.0—0.2) | 0.0—0.6 |  | 0.2 (0.1—0.4) | 0.0—0.8 | 0.0849 |
| APRI | 0.39 (0.30—0.50) | 0.21—0.93 |  | 0.43 (0.34—0.59) | 0.21—1.17 | 0.0483 |
| FIB-4 index | 1.3 (1.0—1.9) | 0.5—3.7 |  | 1.6 (1.1—2.0) | 0.6—4.3 | 0.0584 |
| NAFLD fibrosis score | -0.987 (-1.768 — -0.253) | -3.139—0.737 |  | -0.522 (-1.160—0.078) | -4.218—1.442 | 0.0145 |
| Platelet count (×10^4^/μL) | 21.8 (19.5—26.1) | 12.2—39.8 |  | 19.6 (16.7—22.5) | 8.8—41.0 | 0.0018 |
| AST (IU/L) | 33 (28—40) | 23—84 |  | 34 (28—41) | 20—68 | 0.6350 |
| ALT (IU/L) | 41 (35—53) | 31—99 |  | 44 (37—56) | 31—96 | 0.3490 |
| GGT (IU/L) | 55 (31—85) | 15—218 |  | 53 (37—82) | 19—197 | 0.6998 |
| Albumin (g/dL) | 4.5 (4.2—4.7) | 3.9—5.3 |  | 4.4 (4.3—4.7) | 3.7—5.1 | 0.5766 |
| Creatinine (mg/dL) | 0.7 (0.6—0.9) | 0.4—1.2 |  | 0.7 (0.6—0.9) | 0.4—2.0 | 0.2254 |
| eGFR (mL/min/1.73m^2^) | 83.7 (70.1—97.4) | 49.6—134.6 |  | 80.5 (61.1—96.7) | 29.4—122.7 | 0.2350 |
| HDL-cholesterol (mg/dL) | 50 (42—63) | 28—91 |  | 53 (43—64) | 24—106 | 0.6133 |
| LDL-cholesterol (mg/dL) | 116 (101—134) | 28—91 |  | 122 (104—146) | 40—222 | 0.3698 |
| Triglyceride (mg/dL) | 146 (112—190) | 64—601 |  | 156 (121—189) | 50—656 | 0.5806 |
| Ureic acid (mg/dL) | 5.3 (4.6—6.2) | 2.5—8.5 |  | 5.4 (4.6—6.1) | 2.5—10.3 | 0.8657 |
| β-hydroxybutyrate (μmol/L) | 46.5 (27.9—68.8) | 13.7—199.0 |  | 54.6 (37.7—77.1) | 10.3—363.0 | 0.1334 |
| ACE inhibitor  (no medication/medication) | 98.2%/1.8%  (56/1) | N/A |  | 94.2%/5.8%  (81/5) | N/A | 0.2358 |
| ARB  (no medication/medication) | 56.1%/43.9%  (32/25) | N/A |  | 60.5%/39.5%  (52/34) | N/A | 0.6070 |
| Direct renin inhibitor  (no medication/medication) | 100.0%/0.0%  (57/0) | N/A |  | 100.0%/0.0%  (86/0) | N/A | N/A |
| Lipid-lowering medication  (no medication/medication) | 50.9%/49.1%  (29/28) | N/A |  | 51.1%/48.9%  (44/42) | N/A | 0.9733 |

Abbreviations: IQR, interquartile range; N/A, not applicable; BMI, body mass index; HbA1c, hemoglobin A1c; HOMA-IR, homeostasis model assessment of insulin resistance; NAFLD, non-alcoholic fatty liver disease; APRI, aspartate aminotransferase-to-platelet ratio index; AST, aspartate aminotransferase; ALT, alanine aminotransferase; GGT, γ-glutamyl transpeptidase; eGFR, estimated glomerular filtration rate; HDL, high-density lipoprotein; LDL, low-density lipoprotein; ACE, angiotensin-converting enzyme; ARB, angiotensin receptor blocker.

Supplementary Table 3. Baseline characteristics of subjects with FIB-4 index >1.3

|  | Placebo | |  | Luseogliflozin | |  |
| --- | --- | --- | --- | --- | --- | --- |
|  | Median (IQR) | Range |  | Median (IQR) | Range | P |
| n | 124 | N/A |  | 207 | N/A |  |
| Age (years) | 66 (61—72) | 40—86 |  | 67 (61—71) | 45—89 | 0.9972 |
| Sex (Male/female) | 70.2%/29.8%  (87/37) | N/A |  | 76.3%/23.7%  (158/49) | N/A | 0.2156 |
| BMI | 24.2 (22.0—26.9) | 17.4—40.5 |  | 24.4 (22.2—26.8) | 16.0—37.8 | 0.8198 |
| HbA1c (%) | 7.8 (7.4—8.2) | 6.7—10.7 |  | 7.7 (7.3—8.2) | 6.8—10.6 | 0.3445 |
| HOMA-IR | 2.0 (1.4—3.5) | 0.3—12.1 |  | 2.1 (1.3—3.2) | 0.3—20.4 | 0.7307 |
| Systolic blood pressure (mmHg) | 130 (123—138) | 96—169 |  | 131 (122—140) | 96—178 | 0.5636 |
| Diastolic blood pressure (mmHg) | 75 (70—81) | 52—97 |  | 74 (68—80) | 50—104 | 0.5877 |
| Fatty liver index | 27.2 (16.9—60.6) | 3.9—98.5 |  | 40.8 (22.1—69.0) | 9.7—97.0 | 0.0997 |
| NAFLD liver fat score | -0.5 (-1.5—0.3) | -2.7—3.5 |  | 0.1 (-0.9—0.7) | -2.8—5.5 | 0.0504 |
| Hepatic steatosis index | 34.7 (31.3—38.2) | 25.0—54.8 |  | 34.4 (31.5—38.0) | 23.1—49.7 | 0.7541 |
| Hepamet fibrosis score | 0.2 (0.2—0.4) | 0.0—0.7 |  | 0.2 (0.1—0.4) | 0.0—0.8 | 0.6460 |
| APRI | 0.34 (0.29—0.43) | 0.17—0.93 |  | 0.34 (0.28—0.44) | 0.14—1.17 | 0.8943 |
| FIB-4 index | 1.8 (1.6—2.3) | 1.3—5.6 |  | 1.9 (1.6—2.3) | 1.3—5.9 | 0.4280 |
| NAFLD fibrosis score | 0.021 (-0.463—0.438) | -1.359—2.001 |  | 0.073 (-0.474—0.604) | -1.446—3.336 | 0.5553 |
| Platelet count (×10^4^/μL) | 18.6 (15.8—20.9) | 9.2—28.2 |  | 18.5 (16.3—20.5) | 7.1—30.7 | 0.7518 |
| AST (IU/L) | 25 (21—31) | 15—63 |  | 25 (21—32) | 14—68 | 0.8833 |
| ALT (IU/L) | 23 (17—30) | 10—99 |  | 23 (16—33) | 7—96 | 0.5381 |
| GGT (IU/L) | 30 (20—47) | 9—218 |  | 33 (23—54) | 6—197 | 0.1036 |
| Albumin (g/dL) | 4.4 (4.2—4.6) | 3.7—5.1 |  | 4.4 (4.3—4.6) | 3.5—5.2 | 0.7462 |
| Creatinine (mg/dL) | 0.8 (0.6—0.9) | 0.4—1.7 |  | 0.8 (0.7—1.0) | 0.4—1.6 | 0.0391 |
| eGFR (mL/min/1.73m^2^) | 73.0 (56.3—87.2) | 33.1—129.5 |  | 67.6 (55.9—81.9) | 32.6—122.7 | 0.0854 |
| HDL-cholesterol (mg/dL) | 55 (44—69) | 27—120 |  | 54 (47—66) | 24—108 | 0.9588 |
| LDL-cholesterol (mg/dL) | 118 (99—134) | 45—187 |  | 117 (100—139) | 40—202 | 0.8503 |
| Triglyceride (mg/dL) | 123 (81—164) | 30—810 |  | 121 (87—164) | 31—656 | 0.7169 |
| Ureic acid (mg/dL) | 5.4 (4.4—6.3) | 2.8—8.5 |  | 5.4 (4.4—6.3) | 0.7—10.3 | 0.7803 |
| β-hydroxybutyrate (μmol/L) | 57.6 (31.9—94.5) | 13.2—699.0 |  | 57.2 (36.0—110.0) | 10.3—507.0 | 0.4735 |
| ACE inhibitor  (no medication/medication) | 95.2%/4.8%  (118/6) | N/A |  | 93.7%/6.3%  (194/13) | N/A | 0.5853 |
| ARB  (no medication/medication) | 54.0%/46.0%  (67/57) | N/A |  | 52.7%/47.3%  (109/98) | N/A | 0.8082 |
| Direct renin inhibitor  (no medication/medication) | 100.0%/0.0%  (124/0) | N/A |  | 97.6%/2.4%  (202/5) | N/A | 0.0812 |
| Lipid-lowering medication  (no medication/medication) | 51.6%/48.4%  (64/60) | N/A |  | 48.8%/51.2%  (101/106) | N/A | 0.6193 |

Abbreviations: IQR, interquartile range; N/A, not applicable; BMI, body mass index; HbA1c, hemoglobin A1c; HOMA-IR, homeostasis model assessment of insulin resistance; NAFLD, non-alcoholic fatty liver disease; APRI, aspartate aminotransferase-to-platelet ratio index; AST, aspartate aminotransferase; ALT, alanine aminotransferase; GGT, γ-glutamyl transpeptidase; eGFR, estimated glomerular filtration rate; HDL, high-density lipoprotein; LDL, low-density lipoprotein; ACE, angiotensin-converting enzyme; ARB, angiotensin receptor blocker.
